# Long-Memory and the Sea Level-Temperature Relationship: A Fractional Cointegration Approach

**DOI:** 10.1371/journal.pone.0113439

**Published:** 2014-11-26

**Authors:** Daniel Ventosa-Santaulària, David R. Heres, L. Catalina Martínez-Hernández

**Affiliations:** 1 División de Economía, Centro de Investigación y Docencia Económicas (CIDE), Mexico City, Distrito Federal, Mexico; 2 Department of Economics, University of Konstanz, Konstanz, Germany; CNRS, France

## Abstract

Through thermal expansion of oceans and melting of land-based ice, global warming is very likely contributing to the sea level rise observed during the 20th century. The amount by which further increases in global average temperature could affect sea level is only known with large uncertainties due to the limited capacity of physics-based models to predict sea levels from global surface temperatures. Semi-empirical approaches have been implemented to estimate the statistical relationship between these two variables providing an alternative measure on which to base potentially disrupting impacts on coastal communities and ecosystems. However, only a few of these semi-empirical applications had addressed the spurious inference that is likely to be drawn when one nonstationary process is regressed on another. Furthermore, it has been shown that spurious effects are not eliminated by stationary processes when these possess strong long memory. Our results indicate that both global temperature and sea level indeed present the characteristics of long memory processes. Nevertheless, we find that these variables are fractionally cointegrated when sea-ice extent is incorporated as an instrumental variable for temperature which in our estimations has a statistically significant positive impact on global sea level.

## Introduction

Coastal erosion, loss of coastal wetlands and increased risks of flooding are some of the negative impacts that increases in the sea-level would have on coastal communities and ecosystems [19]. Although the economic costs derived from some of these impacts have been found to be relatively small in terms of GDP losses [1], it is nevertheless important to improve the confidence on the estimates of the relationship between global average temperature and global sea level. Current physics based-models have been shown to be able only to partially replicate recent sea level rise observations based on global average temperature and semi-empirical approaches have been implemented with the intention of filling the predictive gaps in the former class of models [26]. However, most of these studies have not considered the potential spurious inference that is likely to be drawn from a regression of two series with strong temporal properties such as global sea level and global temperature [28]. Two noteworthy exceptions are [29] and [13]. The former performs a cointegration analysis to correct for potential spurious effects and the latter adjusts a nonstationary model through the Kalman filter. However, while a statistically significant impact of sea level on temperature is found in [29], statistical significance for the converse causal relationship is not obtained, which has important implications for climate change adaptation policies. Our study extends the work of Schmith et al. [29] (SJT12 hereinafter) in two ways. First, we obtain fractional orders of integration for the series and present evidence of a long-run relationship between sea level and temperature that is fractionally cointegrated. Second, through the use of instrumental variables we obtain a consistent estimate of the impact of global average temperature on global sea level.

When the distant past of a series affects its current levels, it is said that the process possess long memory. Granger and Joyeaux [11] formally introduced the related concepts of long memory and fractional integration into the field of econometrics. Unit root tests are frequently used to detect the nonstationarity of a series, however, such tests are ill-suited in determining whether a series presents long memory (see, for instance [8], [9]). Tsay and Chung [35] show that the presence of long memory, even in stationary series, leads to spurious relationships. Therefore, determining the fractional order of integration is crucial if valid inferences are to be made regarding the statistical relationship between time series.

Although the approach taken in SJT12 has been long recognized as an appropriate mechanism to correct for the nonstationarity of the series in a regression context, the study does not incorporate more recent developments regarding the implications of long memory in series that seem to be nonstationary. As mentioned above, our study extends the work of SJT12 to the fractionally integrated case allowing us to make statements about the long memory properties of the series and their implications in terms of statistical inference.

Based on our estimates both global temperature and sea level indeed present the characteristics of long memory processes. We however find evidence that supports the fractional cointegration of these two series when sea-ice extent is incorporated as an instrument for sea level to explain global temperatures (hereinafter the term *instrument* refers to a specific instrumental variable while *instrumental variables* or IV, refers to the estimation technique; see [3] (Ch. 4) for an overview of the method). The purpose of including an instrument for temperature is the inconsistency that would result from estimating ordinary least squares (OLS) due to the simultaneity between sea level and temperature, since as empirically shown in SJT12, the former also affects the latter. Sea-ice extent is believed to be a valid instrument since it does not affect sea-level but it is affected by global temperatures. Simulated finite-sample evidence shows that, unlike OLS, inference drawn using IV eliminates the endogeneity bias with fractionally-cointegrated series.

Our results show that global average temperature has a positive impact on global sea level. Importantly, we find evidence of the long-range dependence of these geophysical variables which has profound implications in the modelling approach of future research on the sea level-temperature relationship: the long memory behavior of these series must be acknowledged and appropriately addressed if statistical inference is to be correctly drawn.

## Materials and Methods

Monthly observations on average global sea level (S) and temperature (T) anomalies for the period 1880 (January)-2009 (December) were respectively obtained from [7] and [16]. This dataset is slightly different to the one used in [26] and SJT12. Initially, we used the same yearly dataset than the previous authors; nevertheless, studies on long memory can hardly be performed using series with one hundred observations. Moreover, we obtained simulated finite sample evidence (see Appendix S1) that shows that inference about long-range properties of a series is unreliable when drawn from such small samples. This result is in line with SJT12, where it is considered that much larger samples are needed to obtain statistically valid results. Data on the mean monthly sea-ice extent (km^2^) in the northern hemisphere (I) from 1880 to 2009 were obtained from Chapman (http://arctic.atmos.uiuc.edu/SEAICE). It is important to mention that sea-based ice exhibits a very strong sinusoidal seasonal pattern, which is why we seasonally adjusted it; the seasonal adjustment is done automatically through the X-12 ARIMA program, included in the statistics/econometrics free software *GRETL*. Given the large sample size, the dataset was split in three parts and each one was processed. Raw data and final dataset are available as supporting information (Data S1).

**Figure 1:**
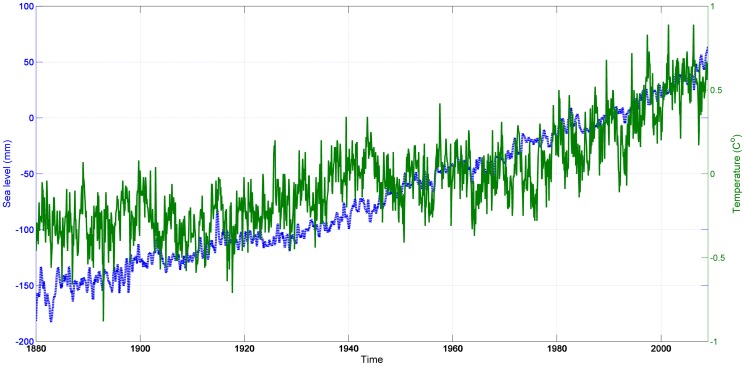
Sea level and global temperature (1880–2009). Continuous line: temperature [7]: global average sea level from satellite altimeter data for 1993–2009 and from coastal and island sea-level measurements from 1880 to 2009. Temperature is zeroed at the 1990 level; dashed line: sea level [16]: GISS data, the average sea level over the 30-year period 1951–1980.

**Figure 2:**
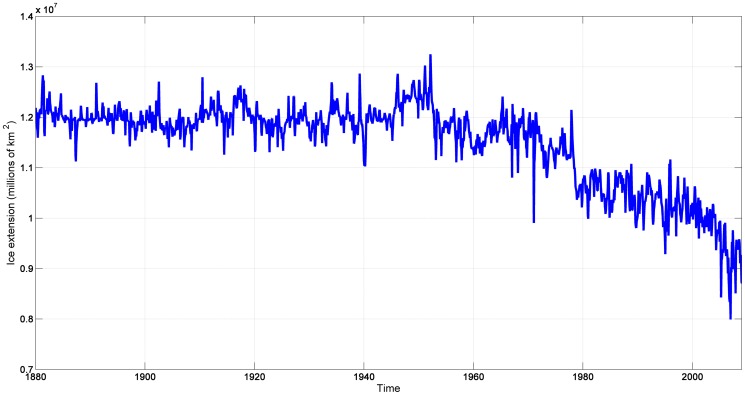
Sea-ice extent (1880-2009) in tenths of millions of *km* ^2^. See http://arctic.atmos.uiuc.edu/SEAICE for details.

The monthly dataset includes 1,560 observations, and, according to our finite sample results (see Appendix S1), we can be confident that our estimates exhibit a negligible bias, contrary to the ones obtained using datasets with only 100 observations. Sea-level and global temperature are depicted in Figure 1 in which a clear upward trend during this period for both series can be observed. Figure 2 shows the downward trend in the sea-based ice series which instruments for temperature in the next section.

**Figure 3:**
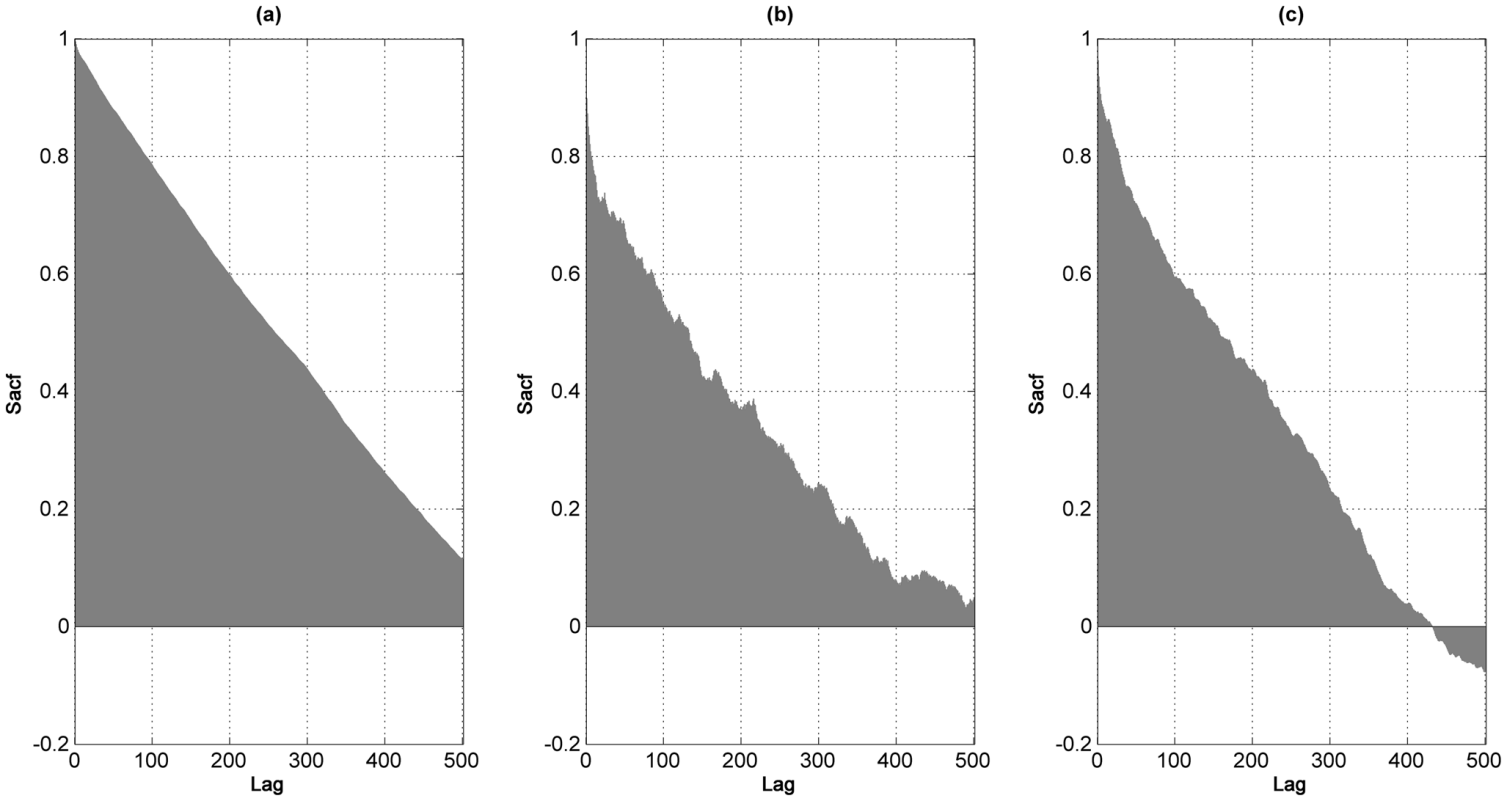
Correlograms for sea level and temperature. Sample Autocorrelation Function (Sacf) for (a) sea-level; (b) temperature; (c) sea-ice extent. Lags in the *x* axis denote which autocorrelation is estimated, whilst the *y* axis measures the value of the autocorrelation. Dashed lines represent the 95% confidence interval; whenever the Sacf falls whithin these limits, the null hyptothesis that the Sacf = 0 cannot be rejected.

Long memory processes represent a bridge between those presenting infinite (nonstationary) and short memory (stationary). The autocorrelation function (Sacf) of processes of the latter kind exhibit exponential decay. Conversely, the Sacf in integrated processes does not decay, whereas the Sacf of long memory processes decays hyperbolically. Figure 3 shows the estimated autocorrelation function up to the 500*^th^* lag of the sea-level, temperature and sea-based ice series; these patterns suggest the presence of long memory in the three series. Formally, a variable 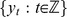 is said to behave as a long memory process if its autocovariances are not absolutely summable, 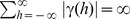, where 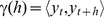 is the 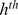 autocovariance. An alternative definition, that can be used as a vehicle to introduce the long memory notation, can be grounded in the hyperbolic decay of the autocovariances, 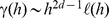 as 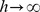, where *d* denotes the long-memory parameter and 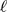 is a slowly varying function [25] (pp. 39–43).

The most common long memory processes are the autoregressive fractionally integrated moving average in which the order of integration includes the interval 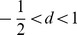, excluding the zero. These processes admit an infinite moving average or an infinite autoregressive process representation [11], [18], [33]. Series are said to be stationary when 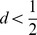 and nonstationary otherwise (when 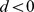, the series are said to be antipersistent). Estimates of a regression between variables that are nonstationary may be spuriously significant. However, there may exist a linear combination that reduces their order of integration measured in the residuals of the estimated regression. In such cases the series are said to be fractionally cointegrated and purged of spurious effects [5], [12], [30]. When the order of integration of the residuals is less than 1/2, deviations from the linear equilibrium relationship between series are slowly–mean–reverting.

Before presenting results in the next section we provide a summary of the semi-empirical statistical procedure we implement to estimate the relationship between temperature and sea-level. Each result is associated with one of the following stages of our procedure and will be presented in the same order in the results section: 1) the strong temporal properties of the variables of interest pose the risk of estimating a spurious regression. Therefore prior to the estimation procedure, we first study whether the series exhibit long-range dependence in their time dynamics by examining the Sacf of the series and formally measuring long memory for each series using the Two-Step Exact Local Whittle estimator (2ELW). To the best of our knowledge, this is the finest available estimator of the persistence of a series and overcomes the limitation of the one-step Local Whittle estimator which is only applicable in the stationary case (i.e., 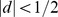) and is inconsistent for 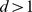 [30]. The 2ELW is consistent and has a normal limit distribution N(0,1/4m) for all values of 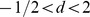, where *m* is the bandwidth parameter of the estimator [31] (Th.3, p. 511). The first step in this estimator is to detrend the data thus avoiding problems originated from time trends. 2) having obtained sound evidence of long-range dependence in the series, we test whether such long-range dependence is statistically the same for the variables in our model. Based on the statistical properties of the 2ELW estimator we perform a test that reveals that S, T, and I statistically have the same time persistence. 3) the latter finding is a necessary condition for fractional cointegration and we estimate the semi-empirical model using the IV method that corrects for the potential endogeneity bias due to simultaneity of temperature and sea-level (i.e., S affects T and viceversa). 4) to ensure that the IV method has been appropriately applied we perform three tests: Sargan's OI test and F test that explore respectively the validity and relevance of the instrument (in this case, I), and a Hausman test that provides evidence on whether there is indeed an endogeneity problem (otherwise OLS would be preferred, because it is an unbiased and more efficient method in that case). The results of these three tests indicate that our instrument is valid and relevant and that the method corrects for the endogeneity bias yielding consistent estimates of the parameters of the semi-empirical model. 5) we implement again the 2ELW estimator to test the persistence of the residuals resulting from the regression and find that the relationship is indeed fractionally cointegrated (this is, there is a linear combination of the variables with a persistence inferior to the one exhibited by the variables). 6) predictions are then produced using our estimated parameters and data.

## Results and Discussion

The first stage from our procedure is to obtain formal evidence of long memory or nonstationarity of the series. The Two-step Exact Local Whittle statistic (2ELW) is estimated for both series. The 2ELW was proposed by Shimotsu and Phillips [30], and Shimotsu [32]; the code is available in Shimotsu's webpage (see http://shimotsu.web.fc2.com/). Based on this estimator, results in Table 1 show that temperature, sea level and sea-ice extent are all nonstationary and present long memory. Note further that the 2ELW estimator is asymptotically distributed as a normal variable, 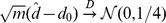, for 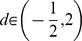 and 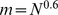, [31] (Th.3, p. 511). This property allows us to build classical 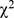 tests, where 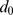 would be the value under 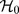. The last row in Table 1 shows the results of a typical inequality test (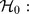 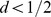).

**Table 1:** Tests of hypothesis on the 2EWL estimator.

Null hypothesis tested	Sea level	Temperature	Sea-based ice
	*d* = 0.677	*d* = 0.618	*d* = 0.636
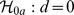 (Wald statistic)	313.858***	261.642***	276.623***
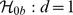 (Wald statistic)	71.188***	99.561***	90.657***
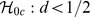 (*t* statistic)	4.639***	3.098***	3.555***

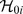
 for i = a,b,c accounts for the null hypothesis being tested.

*** denotes rejection of the null hypothesis at 1% level. *m* = *N* ^7/10^. Hypothesis tests are based on the 2ELW estimate of *d*. The variables have been linearly detrended.

Note that, for the three variables considered in this study, the null hypothesis that 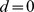 as well as that for 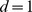 are rejected at the one percent level. Also, for the three variables, the order of integration seems to be superior to 1/2, which implies that they behave as long-range nonstationary variables.

The correlation between the original series, 0.833 (p-value<0.001) is similar to that found in [26] (0.88). However, as noted in [28], this correlation does not take into account the potential stochastic trend in the series which could result in a spurious relationship. The corrected correlation obtained with the filtered series, 0.128 (p-value<0.001), is much weaker (but still significant) than that found between the original nonstationary series.

In order to explore the possibly fractional cointegrated relationship, we first need to show that the sea-level and temperature have, statistically, the same order of integration (this is stage 2 from our procedure). We therefore test the null hypothesis 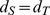. Under the null hypothesis, the test statistic is distributed as a 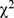 with one degree of freedom. The resulting test statistic is 2.374 and the corresponding p-value is 0.123 which does not allow rejection of the null that both variables have the same order of fractional integration, even at the 10% level. This is also true for the hypothesis 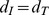 (test statistic is equal to 0.209, with a p-value of 0.648). We therefore have evidence that the three variables share the same fractional integration order.

The results reported in Table 2 correspond to the third stage from our procedure. These are based on an IV regression in which the contemporaneous value as well as two leads of the sea-ice extent series serve as instruments for temperature. We implement this method in order to overcome the potential bias resulting from the simultaneity between the variables sea-level and temperature. In Appendix S1, a finite sample simulation experiment shows that the IV estimates converge to the true parameter values as the sample size grows. Such convergence cannot be attained using OLS. In other words, if sea-level and temperature are nonstationary long-range dependent endogeneous variables belonging to a system of simultaneous equations, then the OLS estimates of the relationship that links these variables would be biased and inconsistent. The IV approach is able to correct such inconsistency, provided that the instrument (sea-based ice) is relevant and valid, and the sample is large enough.

**Table 2:** IV Regression (dependent variable: sea level).

Variable	Coefficient	Standard error	z-statistic	p-value
Constant	−61.956	2.083	−29.750	0.000
Temperature	217.142	8.623	25.180	0.000
*R* 2	0.694			
**Additional Tests:**			**Test statistic**	**p-value**
Sargan OID test^1^			2.627	(0.262)
Weak instruments test^2^			144.482	(<0.001)
Hausman test^3^			470.711	(<0.001)

Heteroskedastic-autocorrelation robust standard errors. *N* = 1,558.

1 Null hypothesis: the instruments are valid.

2 Null hypothesis: the instruments are weak.

3 Null hypothesis: Temperature and innovations are not independent.

Present and future levels of sea-ice extent could be considered valid instruments since they should not be correlated with sea level (validity) except indirectly through their relation with temperature (relevance). Tests on the relevance and validity of sea-ice extent as an instrument for temperature are also reported in Table 2 (stage 4 from our procedure). The instruments are valid and relevant according to the Sargan over-identification (OID) and the weak-instruments robust F-test. Furthermore, the rejection of the null under the Hausman test suggests the presence of endogeneity in which case our IV procedure is not only valid but also advisable. It should be noted, however, that the behavior of these three tests, OID, Hausman and weak-instrument, has not been studied for long-range dependence series and its interpretation should be made with caution.

Predictions obtained from our model of sea-level rise given temperature increases require the series to be fractionally cointegrated. The results from stage 5 from our procedure provide support for this condition. Based on the 2ELW estimator, the residuals from the regression (R hereinafter) in Table 2 have an order of integration of 0.475. We fail to reject the null hypothesis that 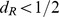 (*t*-test statistic: −0.652; p-value: 0.257) while the hypotheses that 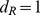, and 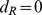 are rejected (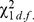 statistics: 188.477 and 154.373, respectively with both p-values inferior to 0.001). Altogether, these results suggest that the series are fractionally cointegrated (i.e., there exists a linear combination of the variables that is integrated of a lower order). In particular, it is important to note that the order of integration of the residuals is inferior to 1/2, which implies that they behave as a stationary process.

The last stage from our procedure is to use the estimates to predict sea-level rises as a consequence of hypothetic increases in global temperatures. Our estimations indicate that temperature has a positive and statistically significant impact on sea level: for each °C increase, sea level would rise by 0.22 m. In the absence of temperature increases, sea level would be reduced by 0.06 m. The magnitude of these impacts are within the range found in [26] where it was estimated a 1.25 m sea level rise for a 5.8°C increase (i.e., 0.22 m per °C). Our estimates are not only within those from other semi-empirical models (see [20], [24] and Table 3) but also within the range predicted from process-based models reported for the four IPCC's AR5 emissions scenarios [20]. The median predictions from these latter class of models are respectively 0.19 m and 0.24 m per °C for the mid and late- 21st century relative to 1986–2005. It is important to note that our estimates cannot incorporate future nonlinear changes in the relationship between temperature and sea level that could occur due to melting of land-based ice at rates that have not been observed in our sample period.

**Table 3:** Results from other semi-empirical models.

Article	Dataset(s)	Methodology	Estimate^1,2,3^	Additional comments
IPCC 2007 [19]	-	-	(3): 0.18–0.59	Benchmark No 1
Rahmstorf (2007) [26]	[6], [15]	SSA and OLS. Additional MA smoothing.	(2): 3.40 (3): 0.50–1.40	First semi- empirical model of SL- T relationship.
Holgate et al (2007) [17]	Same as [26]	Same as [26] but dataset split in four relevant epochs. Does not MA smooth series	(2): 1st half: 8.26 2nd half: 6.60	Critique to [26] on the grounds of nonlinearity of the SL-T relationship and the resulting degrees of freedom due to MA smoothing
Schmith et al (2007) [28]	Same as [26]	Same as [26] but controlling for the trending mechanism.	(2): 5.80	Critique to [26] on the ground of uncontrolled nonstationarity in SL and T.
Rahmstorf (2007b) [27]	Same as [26]	Same as [26] but uses part of the sample (up to 1940) to estimate the T-SL relationship and the rest of the sample for forecasting.	(2): 4.20 (3): 0.93	Reply to critiques in [17] and [28] arguing that the SL-T relationship holds the predictive test.
Vermeer & Rahmstorf (2009) [36]	Same as [26]	Same as [26] but including a time trend in the regression.	(1): 2.50 (+/−0.5) (2): 0.80 (+/−0.17) (3): 0.75–1.90	-
Grinsted et al (2010) [14]	[23], [21], [2], [22]	Monte Carlo inversion (no SSA smoothing).	(2): 6.30 (+/− 1.1) 8.20 (+/− 1.1) 3.00 (+/− 1.8) (3) 0.62–1.60 0.96–2.15 0.30–1.59	Alternative semi-empirical model with longer datasets.
Schmith et al (2012) [29]	[16], [6]	Cointegration analysis between SL and T controlling for other external radiating forces, such as atmospheric CO2 concentration.	None (see comments)	Confirm stochastic trends in variables and a cointegrated long-term relationship. However, the statistical causality is reversed: T (and not SL) adjusts to hold the long-term relationship. They hypothesize that ocean heat capacity, being larger than atmosphere heat capacity, lies at the heart of the difference.
Grassi et al (2013) [13]	Same as [26]	State space model (no SSA smoothing).	(2): 4.56 (3): 0.15–1.50	Alternative semi-empirical model capable of conveniently treating the nonstationary nature of the series.
IPCC 2013 [20]	-	-	(3): 0.28–0.98	Benchmark No 2 (relative to 1986–2005)
Cazenave et al (2014) [4]	Satellite altimetry based global mean sea level (GMSL)	Thermosteric time series: high-pass filter (removes signals years); linear etrendring. Removal of annual and semi-annual signals by fitting 12- and 6-month period; four-month MA smoothing to all series.	(1): 3.30 (+/−0.4)	No semi-empirical model but corrects GMSL time series of SL by removing the inter-anualvariability mostly due to the exchange of water water between oceans, atmosphere and continents.
This work	[7], [16]	Fractional cointegration analysis between SL and T through IV, using sea-based ice as instrument.	(3): 0.22–0.81	Much in line with [29], but (a) the (co)integration is allowed to be of non-integer order to control for possible long memory; (b) we take into account [29]'s eversed causality evidence and estimate the relationship using IV to control for possible endogeneities. Projected SLR based on regression estimates from this study and the means of global mean surface temperature variations from the four scenarios in [20].

(1) mm/year; (2) mm/year/C; (3) SLR: Sea level rise (meters) in 2100.

SSA: Singular spectrum analysis; MA: Moving average; OLS: Ordinary least squares; SL: Sea level; T: Temperature; IV: Instrumental variables.

This study provides a consistent estimate of the impact of temperature on sea level. Our results suggest that sea level would rise by roughly 0.22 m per °C increase. This estimate together with the mean predictions for global temperature increases from IPCC's AR5 four emissions scenarios by the end of the 21st century (1 to 3.7°C) would result in a global sea level rise between 0.22 and 0.81 m. However, as with other semi-empirical estimates, our predictions are not capable of incorporating potentially nonlinear effects deriving from land-based ice melting. Therefore, at the global level our estimates should be taken as a lower-bound impact of temperature on sea level rises that will ultimately inflict severe damages on coastal communities and ecosystems. Furthermore, the melting of ice sheets, among other factors, is expected to impact sea level changes differently across regions [34]. Future research should incorporate recent advances in fractional panel cointegration methods [10] in combination with regional series to examine the impact of temperature variations on regional sea level changes.

Importantly, our results suggest that geophysical variables, such as global temperature, sea level and sea-based ice, behave as long-range dependence processes. Although their nonstationarity became obvious for many researchers their long-lasting relation with their own past has not been considered in empirical research. Taking into account such a property is a capital issue if valid inferences from semi-empirical methods are to be drawn.

## Supporting Information

Appendix S1 Finite sample evidence.
(PDF)

Code S1 Monte Carlo code for Matlab.
(ZIP)

Data S1 Raw data and final dataset.
(ZIP)

Data and Code S1 Data and GRETL code used in regression.
(ZIP)
