# Supplementary material for: Long-Memory and the Sea Level-Temperature Relationship: A Fractional Cointegration Approach
Source: PLoS One. 2014 Nov 26;9(11):e113439. doi: 10.1371/journal.pone.0113439 (PMC4245127; doi:10.1371/journal.pone.0113439)
Supplement: Appendix S1 — Finite sample evidence. (PDF) [file pone.0113439.s001.pdf]

Appendix S1. Finite sample evidence

In this appendix we illustrate the potential gains that can be obtained from Instrumental Variables (IV) estimation, instead of Ordinary Least Squares (OLS). Following the rationale explained in Material and Methods, neither sea-level nor temperature, are exogenous, while ice affects sea-level only through its relationship with temperature. The set of equations that mimics this hypothesis is the following: first, we define the innovations: $u_{1,t} \sim ARFIMA(p, \frac{1}{4}, 0)$, $u_{2,t} \sim ARFIMA(p, \frac{1}{6}, 0)$, and $u_{z,t} \sim AR(p)$. The three noises are independent of each other. We allow for two possibilities: either $p = 0$ (no short-memory autocorrelation), or $p = 1$ (the short memory behaves as a stationary autoregressive process of order one, with $\phi_1 = 0.7$, $\phi_2 = 0.5$, and $\phi_z = 0.3$, respectively. These innovations are then used to build the data-generating processes (dgp) of sea-level (S_t), temperature (T_t), and ice (I_t):

$$\begin{aligned} S_t &= \alpha_S + \beta_S T_t + u_{1,t}, & (1)\\ T_t &= \alpha_T + \beta_T u_{1,t} + u_{2,t}, & (2)\\ I_t &= \alpha_I + \gamma u_{2,t} + u_{z,t}. & (3) \end{aligned}$$

Note that, on the one hand, S_t and T_t form a simultaneous equation system: both variables include $u_{1,t}$ in their dgp; they are therefore endogenously defined. On the other hand, ice is directly related to temperature (through $u_{2,t}$). This equation allows I_t to be an adequate instrument; it is related to temperature, but not to the innovations of sea-level.

Equations 1, 2, and 3 are then used to generate simulated processes of S_t, T_t, and I_t, respectively. The simulated processes are then used to estimate β_S of the specification $S_t = \alpha_S + \beta_S T_t + u_{1,t}$. We repeat this R times (the number of replications) for different sample sizes ($T = 100, 250, 500, 1000, 1560, 2500, 5000$). We estimate the latter using four formulas:

1. OLS, $\vec{\beta}_{OLS} = (X'X)^{-1}X'y$,
2. IV with one instrument (I_t), $\vec{\beta}_{IV} = (Z'X)^{-1}Z'y$,
3. IV with two lagged instruments (I_t, I_{t-1}, and I_{t-2}),

$$\vec{\beta}_{IV^{bis}} = (X'Z(Z'Z)^{-1}Z'X)^{-1}X'Z(Z'Z)^{-1}Z'y.$$

4. IV with two lead instruments, $\vec{\beta}_{IV^{tris}}$ (I_t, I_{t+1}, and I_{t+2}). The formula is the same as above, only the instrument in Z are different.

The vector y includes the observations of sea-level, the matrix X includes the vector of ones (the constant term) in the first column and the observations of temperature in the second column. For the second estimator, the matrix Z includes the vector of ones in the first column and the contemporaneous observations of sea-based ice in the second column. For the third estimator, it includes the vector of ones, the contemporaneous observations of sea-based ice, the one-period lagged observations of sea-based ice, and the two-periods lagged observations of sea-based ice, in the first, second, third and fourth columns, respectively. Finally, the fourth estimator, is the same as the third one, only instead of lags of ice, we use leads of it. In both the third and fourth estimators two initial (final) observations are lost because of the lags (leads). Table S1A presents an estimate of the bias for each estimator, as well as its estimated standard deviation, below in parenthesis. The first four columns show estimates of the bias and standard deviation under innovations with no short-memory component, whilst the data simulated for the last four columns include the short-memory AR(1) term. The parametric setting is summarized in Table S1B.

Table S1A. OLS and IV estimates of β. Bias and Standard Deviation.

Sample Size	White noise innovations				Autocorrelated innovations			
	OLS	IV	IVbis	IV^{tris}	OLS	IV	IVbis	IV^{tris}
100	0.355	-0.023	-0.014	-0.014	0.623	-0.022	0.009	0.009
	(0.126)	(0.223)	(0.236)	(0.237)	(0.064)	(0.227)	(0.226)	(0.225)
250	0.356	-0.018	-0.017	-0.016	0.624	-0.018	-0.007	-0.007
	(0.127)	(0.218)	(0.233)	(0.233)	(0.060)	(0.201)	(0.214)	(0.214)
500	0.361	-0.013	-0.013	-0.013	0.626	-0.014	-0.010	-0.010
	(0.107)	(0.187)	(0.199)	(0.199)	(0.051)	(0.168)	(0.183)	(0.183)
1,000	0.366	-0.010	-0.010	-0.010	0.629	-0.007	-0.004	-0.004
	(0.08)	(0.142)	(0.151)	(0.151)	(0.039)	(0.128)	(0.141)	(0.141)
1,560	0.369	-0.005	-0.005	-0.006	0.629	-0.005	-0.004	-0.004
	(0.065)	(0.117)	(0.124)	(0.124)	(0.032)	(0.105)	(0.116)	(0.116)
2,500	0.371	-0.004	-0.004	-0.004	0.630	-0.002	-0.001	-0.001
	(0.052)	(0.094)	(0.1)	(0.100)	(0.025)	(0.083)	(0.092)	(0.092)
5,000	0.373	-0.001	-0.001	-0.001	0.631	-0.001	-0.001	-0.001
	(0.036)	(0.066)	(0.071)	(0.070)	(0.018)	(0.060)	(0.067)	(0.067)

Bias= $R^{-1}\sum_{i=1}^{R}\hat{\beta}_w - \beta_S$, Standard deviation = $(R-1)^{-1}\sum_{i=1}^{R}(\hat{\beta}_w - \beta_S)^2$, for $w = OLS, IV, IV^{bis}, IV^{tris}$.
Replications: $R = 10,000$.

The bias of OLS estimates does not fade out as the sample size grows; it remains approximately around 0.37 (more than 200%). Conversely, IV estimates exhibit a small bias, even for small samples such as 100 observations (15%) that vanishes as the sample grows (less than 0.5% for samples larger than 1500 observations). Moreover, its standard deviation diminishes consistently. The previous remarks remain valid whether the innovations have an AR component or not. It is noteworthy to mention that overidentified IV estimates perform slightly better than exactly identified IV. Finally, results do not vary whether lags or leads are employed as instruments.

Table S1B. Parameter setting of the Monte Carlo.

$\alpha_S = -1.70$	$\alpha_I = 1.20$	$\alpha_T = -0.50$	$\beta_S = 0.170$	$\beta_T = 0.45$
$\gamma = -2.50$	$\phi_S = 0.70$	$\phi_I = 0.30$	$\phi_T = 0.50$	$\sigma_S = 0.70$
$\sigma_I = 0.80$	$\sigma_T = 0.50$			

It is important to stress the fact that this simulation experiment exhibits evidence based on a specific parametric setting. Although we made a considerable number of variations in which the IV estimates systematically outperforms OLS and there is always evidence of convergence for the former, and non convergence for the latter, the results naturally vary (the code of the Monte Carlo experiment written in *Matlab* is available as supplementary material; see Code S1). Further theoretical work on this matter is required, however this is out of the scope of the present study.
