# Supplementary material for: Long-Memory and the Sea Level-Temperature Relationship: A Fractional Cointegration Approach
Source: PLoS One. 2014 Nov 26;9(11):e113439. doi: 10.1371/journal.pone.0113439 (PMC4245127; doi:10.1371/journal.pone.0113439)
Supplement: Code S1 — Monte Carlo code for Matlab. (ZIP) [file pone.0113439.s002.zip › Code S1/Readme.pdf]

Instructions

There are six files (the first two are intended to check the robustness of the long memory simulator; the third file generates the Monte Carlo results; the remaining files are necessary additional files):

1. **Simu_1**: Simulator of ARFIMA processes using Simone Fatichi's code. This code simulates the process for sea level, temperature and Ice, and then estimates the relationship using OLS and IV (with only 2 instruments). For the OLS estimation, please download J.P.Lesage's toolbox: http://www.business.txstate.edu/users/jl47/
2. **Simu_2**: Simulator of ARFIMA processes using Shimotsu's code. This code simulates the process for sea level, temperature and Ice, and then estimates the relationship using IV (with only 2 instruments).
3. **Simu_3**: Simulator of ARFIMA processes using Shimotsu's code. This code simulates the process for sea level, temperature and Ice, and then estimates the relationship using IV.
4. **ARFIMA_SIM**: Simone Fatichi function, simulates ARFIMA(p,d,q) processes.
5. **fracdiff**: Shimotsu's function, simulates I(d) processes
6. **sigmad:** function, computes the value of the variance (input of the ARFIMA simulator)
