# Supplementary material for: Long-Memory and the Sea Level-Temperature Relationship: A Fractional Cointegration Approach
Source: PLoS One. 2014 Nov 26;9(11):e113439. doi: 10.1371/journal.pone.0113439 (PMC4245127; doi:10.1371/journal.pone.0113439)
Supplement: Data S1 — Raw data and final dataset. (ZIP) [file pone.0113439.s003.zip › Data S1/Readme.pdf]

Instructions

There are five files:

1. **DataM_CC1922**: Gretl file; contains the Ice series, the seasonally adjusted series, and the trend series that X-12 ARIMA produces, subperiod 1880:01 – 1922:12
2. **DataM_CC1965**: Gretl file; contains the Ice series, the seasonally adjusted series, and the trend series that X-12 ARIMA produces, subperiod 1923:01 – 1965:12
3. **DataM_CC2009**: Gretl file; contains the Ice series, the seasonally adjusted series, and the trend series that X-12 ARIMA produces, subperiod 1966:01 – 2009:12
4. **Seasonality_iCE**: X-12 ARIMA Output, Tables D 8.A (seasonality tests for each subperiod).
5. **Final_Dataset**: Excel file; contains the three variables (Sea level, Temperature and Seasonally adjusted Ice) plus the two leads of Ice, used as instruments.

The Gretl files can be opened using the freeware GRETL: (http://gretl.sourceforge.net/index.html). You also have to download the X-12 ARIMA add-in (in the same webpage as above). To seasonally adjust a series, select the series (one "clic"), then go to the "Variable" Menu and clic "ARIMA-X-12".
