# Supplementary material for: Long-Memory and the Sea Level-Temperature Relationship: A Fractional Cointegration Approach
Source: PLoS One. 2014 Nov 26;9(11):e113439. doi: 10.1371/journal.pone.0113439 (PMC4245127; doi:10.1371/journal.pone.0113439)
Supplement: Data and Code S1 — Data and GRETL code used in regression. (ZIP) [file pone.0113439.s004.zip › Data and Code S2/Readme.pdf]

Instructions

There is only one file, GretlDATASET. The Gretl files can be opened using the freeware GRETL: (http://gretl.sourceforge.net/index.html). To obtain the estimates referred in Table 5, follow the instructions:

1. Open the file in Gretl:

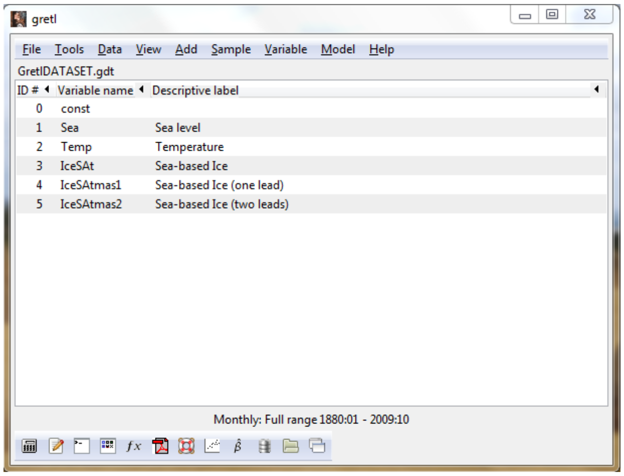

2. Go to <Model> and select <Instrumental Variables> then Two-Stage Least Squares. You should get to the following Menu:

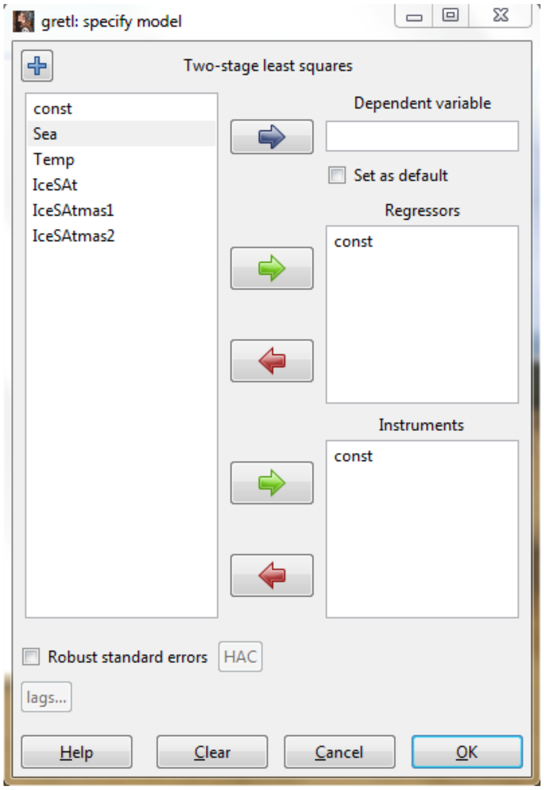

3.- Use the arrow to set "Sea" as the dependent variable, "Temp" as the Regressor (in addition to the constant term) and IceSAt, IceSAtmas1, and IceSAtmas2, as instruments (again, in addition to the constant term). Also, check the "Robust standard errors" box. The Menu should look like this:

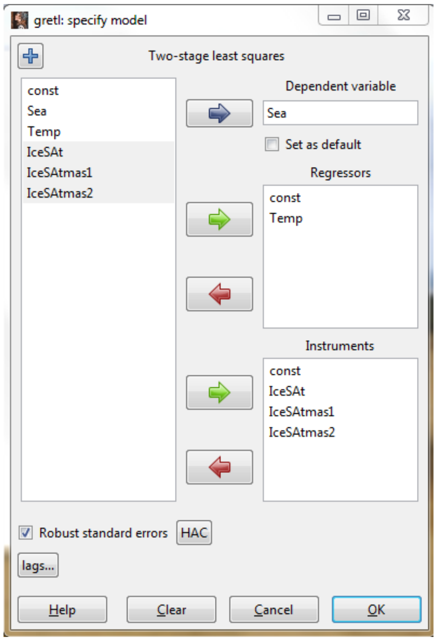

4.- When you click <OK>, you should get exactly the same results as in Table 5:

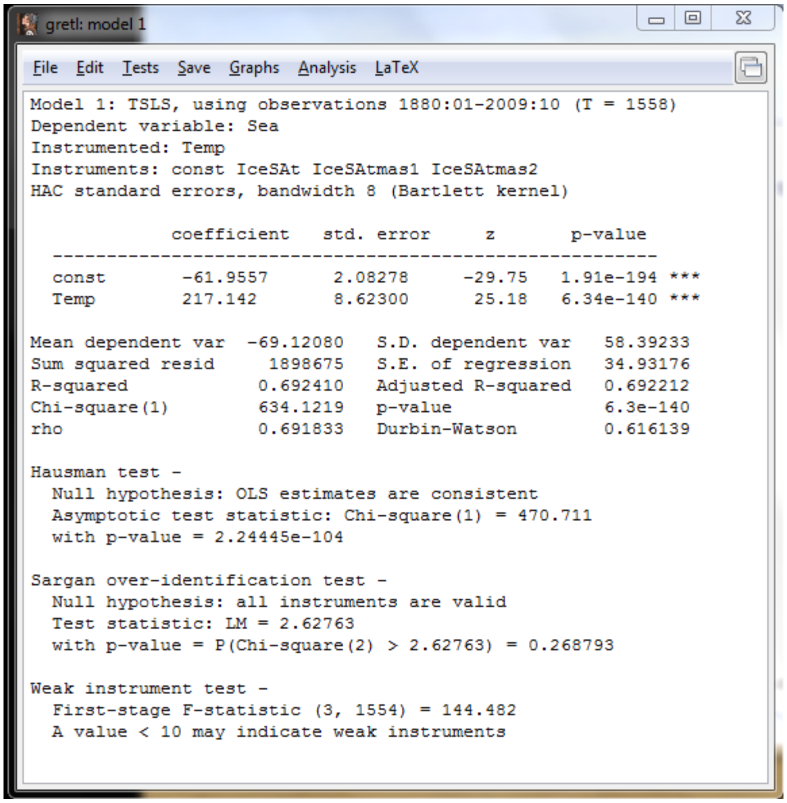


```
Model 1: TSLS, using observations 1880:01-2009:10 (T = 1558)
Dependent variable: Sea
Instrumented: Temp
Instruments: const IceSAt IceSAtmas1 IceSAtmas2
HAC standard errors, bandwidth 8 (Bartlett kernel)

             coefficient   std. error     z       p-value
  ---------------------------------------------------------
  const       -61.9557     2.08278     -29.75   1.91e-194 ***
  Temp        217.142      8.62300      25.18   6.34e-140 ***

Mean dependent var  -69.12080   S.D. dependent var   58.39233
Sum squared resid    1898675    S.E. of regression   34.93176
R-squared           0.692410    Adjusted R-squared   0.692212
Chi-square(1)       634.1219    p-value              6.3e-140
rho                 0.691833    Durbin-Watson        0.616139

Hausman test -
  Null hypothesis: OLS estimates are consistent
  Asymptotic test statistic: Chi-square(1) = 470.711
  with p-value = 2.24445e-104

Sargan over-identification test -
  Null hypothesis: all instruments are valid
  Test statistic: LM = 2.62763
  with p-value = P(Chi-square(2) > 2.62763) = 0.268793

Weak instrument test -
  First-stage F-statistic (3, 1554) = 144.482
  A value < 10 may indicate weak instruments
```
